# Bacterial membrane vesicles as effective immunotherapeutic agents in melanoma, colon, and breast cancer

**DOI:** 10.20517/evcna.2026.07

**Published:** 2026-07-06

**Authors:** Jan Lötvall, Negar Ordouzadeh, Kyong-Su Park

**Affiliations:** ^1^Krefting Research Centre, Institute of Medicine, Sahlgrenska Academy, University of Gothenburg, Gothenburg 40530, Sweden.; ^2^Exocure Sweden AB, Gothenburg 41126, Sweden.

**Keywords:** Synthetic bacterial vesicles, outer membrane vesicles, melanoma, colon cancer, breast cancer, intratumoral immunization, local immunization, cancer immunotherapy

## Abstract

**Aim:** Bacterial outer membrane vesicles are highly immunogenic due to their native bacterial antigens, however，their toxic components trigger systemic inflammation. We previously developed non-toxic synthetic bacterial vesicles (SyBV), and confirmed their anti-tumor effects when co-administered with tumor antigens. Building on this, the present study aimed to extensively assess the immunotherapeutic potential of local SyBV administration, leveraging endogenous tumor antigens across multiple cancer models.

**Methods:** SyBV were generated from *Escherichia coli* membranes via lysozyme-induced cell lysis and ionic stress. Multiple intratumoral administrations of SyBV at different doses were evaluated in melanoma-bearing mice. Therapeutic efficacy was further assessed according to initial tumor size and across multiple tumor models, including melanoma, colon, breast, kidney, and lung cancers. Systemic anti-tumor effects were also examined by evaluating tumor growth at distant, non-injected tumor sites following local SyBV immunization.

**Results:** High-dose SyBV treatment effectively suppressed tumor growth in melanoma-bearing mice. Anti-tumor efficacy varied depending on initial tumor size, with the strongest effects observed when treatment was initiated at smaller tumor volumes. Local SyBV immunization also induced systemic anti-tumor responses, reducing tumor growth at distant tumor sites. Furthermore, SyBV demonstrated significant immunotherapeutic efficacy in colon and breast cancer models, but not in kidney or lung cancer models, highlighting their therapeutic potential in selected cancer types.

**Conclusion:** Our findings show that engineered bacterial vesicles serve as effective standalone cancer immunotherapeutics, allowing simpler drug formulations and potentially improving their clinical translation.

## INTRODUCTION

Cancer remains a leading global cause of death, with ~19.3 million new cases and nearly 10 million deaths annually^[1]^. While chemotherapy remains widely used, cancer vaccines are being developed to enhance immune recognition of tumors^[2]^. Central to these vaccines are adjuvants, which boost the magnitude and durability of immune responses. However, adjuvant innovation has progressed slowly^[3]^. Most approved adjuvants are aluminum-based, with limitations such as low bioavailability and immunotoxicity, which restrict safe dosing and consistent therapeutic outcomes across patients^[4,5]^.

Outer membrane vesicles (OMV) are nanoscale particles released by bacteria that carry proteins, lipids, and nucleic acids^[6]^. They can deliver antigens to dendritic cells and elicit strong adaptive immune responses by activating T-lymphocytes, making them attractive as vaccine adjuvants^[7]^. Their immune-stimulatory effects have also been observed in cancer models, highlighting their potential for cancer immunotherapy development^[8]^. However, OMV can also induce adverse effects, such as sepsis or lung injury, posing a major challenge to their clinical application^[9,10]^.

To address the challenges of using OMV in clinical settings, we previously developed detoxified OMV-like vesicles from bacterial membranes, termed synthetic bacterial vesicles (SyBV)^[11]^. Unlike natural OMV, these engineered vesicles exhibit no unintended toxicity, even at high doses. Moreover, systemic administration of SyBV, combined with exogenous tumor antigens, has been shown to strongly activate both humoral and cellular adaptive immune responses, leading to specific anti-tumor effects when combined with anti-PD-1 immunotherapy in melanoma^[11]^. In the present study, we advance this prior work by hypothesizing that local administration of SyBV alone is sufficient to suppress tumor growth across multiple murine solid tumor models, without requiring exogenous tumor antigens, as endogenous tumor antigens are naturally present within tumor. To test this hypothesis, SyBV were generated through a series of biochemical processes from *Escherichia coli* (*E. coli*) membranes and then intratumorally injected in mice carrying melanoma or colon-, breast-, lung-, or kidney cancer tumors.

## METHODS

### Animals

Wild-type C57BL/6 mice or Balb/c mice, aged 5-7 weeks, were purchased from Charles River and kept at Experimental Biomedicine (EBM) at the University of Gothenburg, Sweden. The research received approval from the local Animal Ethics Committee in Gothenburg, Sweden (permit no. Dnr 5.8.18-10595/2023). Some experiments were approved by the Axis Bioservices Animal Welfare and Ethical Review Committee in the United Kingdom (Project license number PPL2862), and all procedures followed the guidelines outlined in the Animal (Scientific Procedures) Act 1986.

### Production of SyBV

*E. coli* cultures were pelleted and resuspended in 20 mM Tris-HCl (pH 8.0) with 20% sucrose, followed by lysozyme (600 µg/g cells) and 0.1 M EDTA (0.2 mL/g cells) treatment. Spheroplasts were pelleted and sonicated in 10 mM Tris-HCl (pH 8.0) at 4 °C using a Q55 Sonicator (QSonica, Newtown, CT). Membranes were isolated by centrifugation at 40,000 × *g* for 1 h at 4 °C, then incubated with 0.5% Sarkosyl (Sigma Aldrich, St. Louis, MO) for 20 min and with 200 mM Na_2_CO_3_ (pH 11) for 1 h at 25 °C to generate outer membrane sheets. Samples were applied to a step gradient (50%, 30%, and 10% iodixanol; Axis-Shield PoC AS, Oslo, Norway) and ultracentrifuged at 100,000 × *g* for 2 h at 4 °C. Fractions between 10% and 30% were collected, sonicated in an ultrasonic bath (Grant, Cambridge, UK), and designated as SyBV.

### Preparation of OMV

OMV derived from *E. coli* were isolated using a previously described protocol with modifications^[11]^. Briefly, bacterial cultures were centrifuged twice at 6,000 × *g* at 4 °C for 20 min, the supernatant was filtered and concentrated, and OMV were collected by ultracentrifugation at 150,000 × *g* for 3 h and resuspended in phosphate-buffered saline (PBS).

### Transmission electron microscopy (TEM)

The isolated SyBV were analyzed by negative staining for TEM. These vesicles were applied to glow-discharged 200-mesh formvar- and carbon-coated copper grids (Electron Microscopy Sciences, Hatfield, PA) for 5 min, rinsed with water, and fixed with 2.5% glutaraldehyde. After two washes in H_2_O, samples were stained with 2% uranyl acetate for 1.5 min. Images were acquired using a Talos L120C transmission electron microscope (Thermo Fisher Scientific, Waltham, MA) operated at 120 kV and equipped with a charge-coupled device (CCD) camera.

### Nanoparticle tracking analysis (NTA)

SyBV were diluted in PBS, and vesicle numbers were quantified using a ZetaView analyzer (Particle Metrix GmbH, Meerbusch, Germany). Analyses were performed in triplicate, with each data point derived from two stationary layers and five measurements per layer. Camera sensitivity was set to 70 for all measurements, and data were processed using ZetaView software version 8.2.30.1.

### Western blotting

Bacterial OMV and SyBV were resolved on a 10% sodium dodecyl sulfate-polyacrylamide gel electrophoresis (SDS-PAGE) gel and transferred onto a polyvinylidene difluoride (PVDF) membrane. After blocking, the membrane was probed with an anti-OmpF antibody or an anti-lipid A antibody (ThermoFisher Scientific, Waltham, MA). The bound antibodies were detected using a horseradish peroxidase-linked secondary antibody, and the signals were visualized by chemiluminescence.

### DNA analysis

DNA from SyBV or OMV was extracted using the Qiamp DNA Blood Mini Kit (Qiagen, Hilden, Germany) following the manufacturer’s instructions. The quality, concentration, and fragment size of 1 µL of the extracted DNA were assessed via capillary electrophoresis with an Agilent High Sensitivity DNA chip on an Agilent 2100 Bioanalyzer (Agilent Technologies GmbH, Berlin, Germany).

### Limulus amebocyte lysate (LAL) assay

The lipopolysaccharide (LPS) level of SyBV or OMV was measured using the LAL chromogenic endotoxin quantification kit (Thermo Fisher Scientific, Waltham, MA) according to the manufacturer’s instructions.

### MTT (3-[4,5-Dimethylthiazol-2-yl]-2,5-diphenyltetrasolium bromide) assay

B16F10 (melanoma) cells were seeded in a 96-well plate (2 × 10^4^ cells per well) and treated with SyBV (10^9^, 3 × 10^9^, or 10^10^ particles/ mL) for 24 h. MTT (Sigma-Aldrich, St. Louis, MO) was added to the cells, followed by incubation with dimethyl sulfoxide to solubilize the formazan crystals. Absorbance was measured at 570 nm with a reference wavelength of 630 nm using a microtiter plate reader.

### *In vivo* tumor experiment

B16F10 (melanoma), EMT6 (breast cancer), LL/2 (Lewis lung cancer), and RENCA (renal cancer) cell lines were obtained from ATCC (Manassas, VA), while the MC38 (colon cancer) cell line was purchased from Sigma-Aldrich (St. Louis, MO). A total of 5 × 10^5^ cells from each mouse cancer cell line were subcutaneously inoculated into the flanks of C57BL/6 or Balb/c mice. Once tumors reached approximately 10-100 mm^3^, mice were randomly assigned to either sham or SyBV-treatment groups. In the B16F10 melanoma model, mice received intratumoral injections of SyBV (10^9^ or 10^10^) five or six times at 3-day intervals. In the other tumor models, SyBV (10^10^) was injected intratumorally five or six times at 3-day intervals. For all models, anti-mouse PD-1 antibody (100 µg; BioXcell, West Lebanon, NH) was administered intraperitoneally 1 day prior to immunization with SyBV. Tumor growth was monitored every 2-3 days with calipers, and tumor volume was calculated as (width)^2^ × (length)/2. Mouse serum was diluted 1:500 in PBS containing 1% bovine serum albumin (BSA) and applied to 96-well plates coated with 200 ng of tumor vesicular proteins. After a 2-h incubation, bound antibodies were detected using a peroxidase-conjugated anti-mouse IgG antibody (Thermo Fisher Scientific, Waltham, MA).

### Schematic illustrations

Created with BioRender.com.

### Statistical analysis

All data are presented as mean ± standard error of the mean (SEM). Independent replication of each tumor experiment was not performed. All data were tested for normal distribution using the Shapiro-Wilk test. For comparisons between two groups, an unpaired two-tailed Student’s *t*-test was used when data were normally distributed with similar variance. Otherwise, a nonparametric Mann-Whitney test was applied. For experiments with multiple groups or two independent variables, two-way analysis of variance (ANOVA) was applied, followed by Tukey’s post hoc test for multiple comparisons when normality and equal variance assumptions were met. When data did not meet normality assumptions, the nonparametric Kruskal-Wallis test was used, followed by Dunn’s post hoc test for pairwise comparisons. Effect sizes and variance estimates were considered to determine whether parametric or nonparametric tests were appropriate. Multiple comparison corrections were applied consistently according to the chosen post hoc tests. A significance level of *P* < 0.05 was considered statistically significant. All the statistical analyses were performed using GraphPad Prism 10 (GraphPad Software Inc., San Diego, CA).

## RESULTS

### Production of highly pure SyBV from *E. coli* membranes

SyBV were efficiently produced from *E. coli* membranes using a stepwise purification procedure [Figure 1A]. Briefly, bacterial pellets were enzymatically digested with lysozyme, followed by sonication in the presence of sarkosyl detergent to disrupt the inner membrane. Subsequent exposure to high pH conditions removed residual cytosolic contents, yielding enriched outer membrane fractions. These fractions were subjected to buoyant density gradient ultracentrifugation (10%-30% iodixanol), which enabled efficient separation from cellular debris and protein aggregates. The resulting outer membrane sheets were converted to SyBV through mild sonication, producing nanosized vesicles with high structural uniformity. NTA [Supplementary Figure 1A] and TEM [Figure 1B] analyses confirmed that SyBV were spherical, with diameters ranging between 50-200 nm (average size: 153.6 ± 1.2 nm; *n* = 3 independent preparations). Importantly, the preparations were devoid of detectable contaminants such as pili-like structures or cell fragments, indicating high purity [Figure 1B]. Moreover, OmpF, a major constituent of the outer membrane implicated in immune responses^[12]^, was identified in SyBV [Supplementary Figure 1B]. SyBV were generated in substantially greater amounts than naturally derived OMV from an equivalent culture volume [Supplementary Figure 1C]. In addition, SyBV exhibited higher purity, as indicated by a greater particle-to-protein ratio [Supplementary Figure 1D] and the absence of DNA contamination [Supplementary Figure 1E], in agreement with the TEM observations. Interestingly, LPS levels in SyBV were found to be comparable to those in OMV [Supplementary Figure 1F and G]. The electron microscopy further confirmed that the structural integrity of SyBV is maintained for at least two weeks, indicating a high degree of stability [Supplementary Figure 1H]. Collectively, these findings demonstrate the successful production of highly pure SyBV, establishing a reliable foundation for subsequent *in vivo* functional studies.

**Figure 1:**
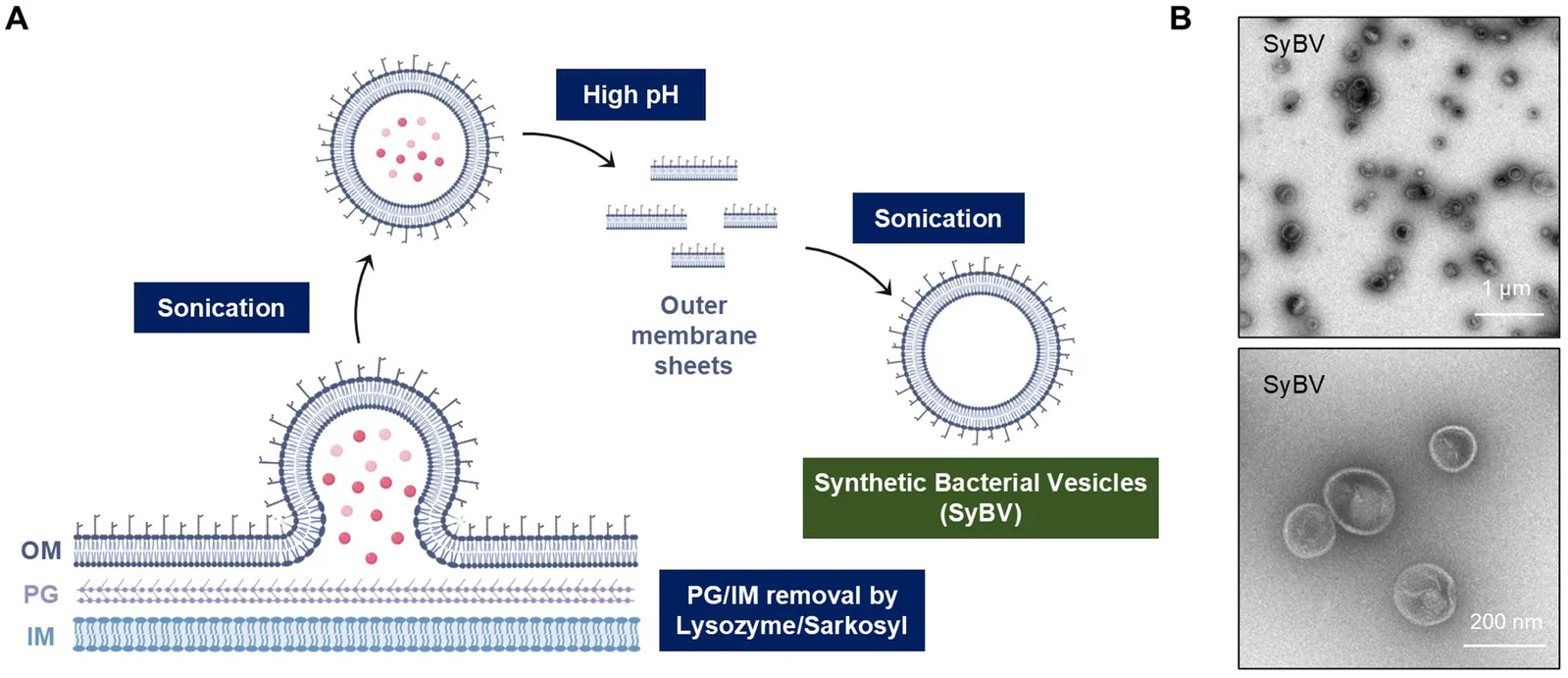
Schematic diagram for SyBV isolation from bacterial membranes. (A) OM sheets were separated from the PG layer and IM through sequential chemical treatments, followed by sonication and high pH treatment (200 mM Na_2_CO_3_, pH 11). The resulting membrane sheets were re-vesiculated into SyBV by mild sonication; (B) TEM images showing the morphology of the isolated SyBV. Scale bars, 1 µm (upper image) and 200 nm (lower image). Created with BioRender.com (2026). SyBV: Synthetic bacterial vesicle; OM: outer membrane; PG: peptidoglycan; IM: inner membrane; TEM: transmission electron microscopy.

### Complete tumor shrinkage in anti-PD-1-resistant melanoma following intratumoral SyBV immunization

We evaluated the anti-tumor efficacy of SyBV in the subcutaneous B16F10 melanoma model, a well-established “cold” tumor that lacks T cell infiltration and is resistant to checkpoint blockade^[13]^. We previously demonstrated that a maximum dose of 2.5 × 10^11^ SyBV caused no changes in body temperature and did not induce systemic inflammation^[11]^. On the basis of these findings, a higher dose of 10^10^ SyBV per administration was selected and given six times. When tumors reached 10-30 mm^3^, mice received intratumoral injections of SyBV at two doses (10^9^ or 10^10^ particles) directly into the tumor, and tumor growth was monitored [Figure 2A]. Individual tumor growth for each mouse from Figure 2A is presented in Figure 2B-E. Anti-PD-1 monotherapy was included to confirm the model’s inherent resistance. As expected, PD-1 blockade alone failed to significantly inhibit tumor growth compared to the sham-treated group [Figure 2B and C]. In contrast, SyBV administration in combination with anti-PD-1 robustly suppressed tumor progression at both doses [Figure 2D and E]. Even at the lower dose (10^9^), SyBV plus anti-PD-1 reduced tumor volume by 92%, (*P* < 0.001 compared to sham) with two of nine mice exhibiting complete regression following repeated treatment [Figure 2D]. The higher dose (10^10^) achieved nearly 99% tumor reduction (*P* < 0.001), and six of nine mice displayed complete tumor clearance by three weeks [Figure 2E]. Moreover, SyBV alone significantly inhibited melanoma growth, consistent with previous reports^[14]^, and the combination of SyBV with an anti-PD-1 antibody exhibited an even more pronounced effect [Supplementary Figure 2]. These results demonstrate that SyBV can convert anti-PD-1-resistant tumors into responsive ones, highlighting its potential as a sensitizing agent for checkpoint blockade. Notably, the higher dose (10^10^) of SyBV did not induce any increase in systemic pro-inflammatory cytokines during either the acute phase (6 h) or the chronic phase (Day 21), whereas OMV provoked substantial cytokine release [Supplementary Figure 3]. Moreover, no local skin reactions were observed in any SyBV-treated mice, even at the higher dose and with repeated administration, indicating a favorable safety profile (Data not shown for representative images). We next examined the viability of melanoma cells treated with SyBV and found no direct effect [Supplementary Figure 4], suggesting that their anti-tumor effects are indirect and likely mediated through immune cells.

**Figure 2:**
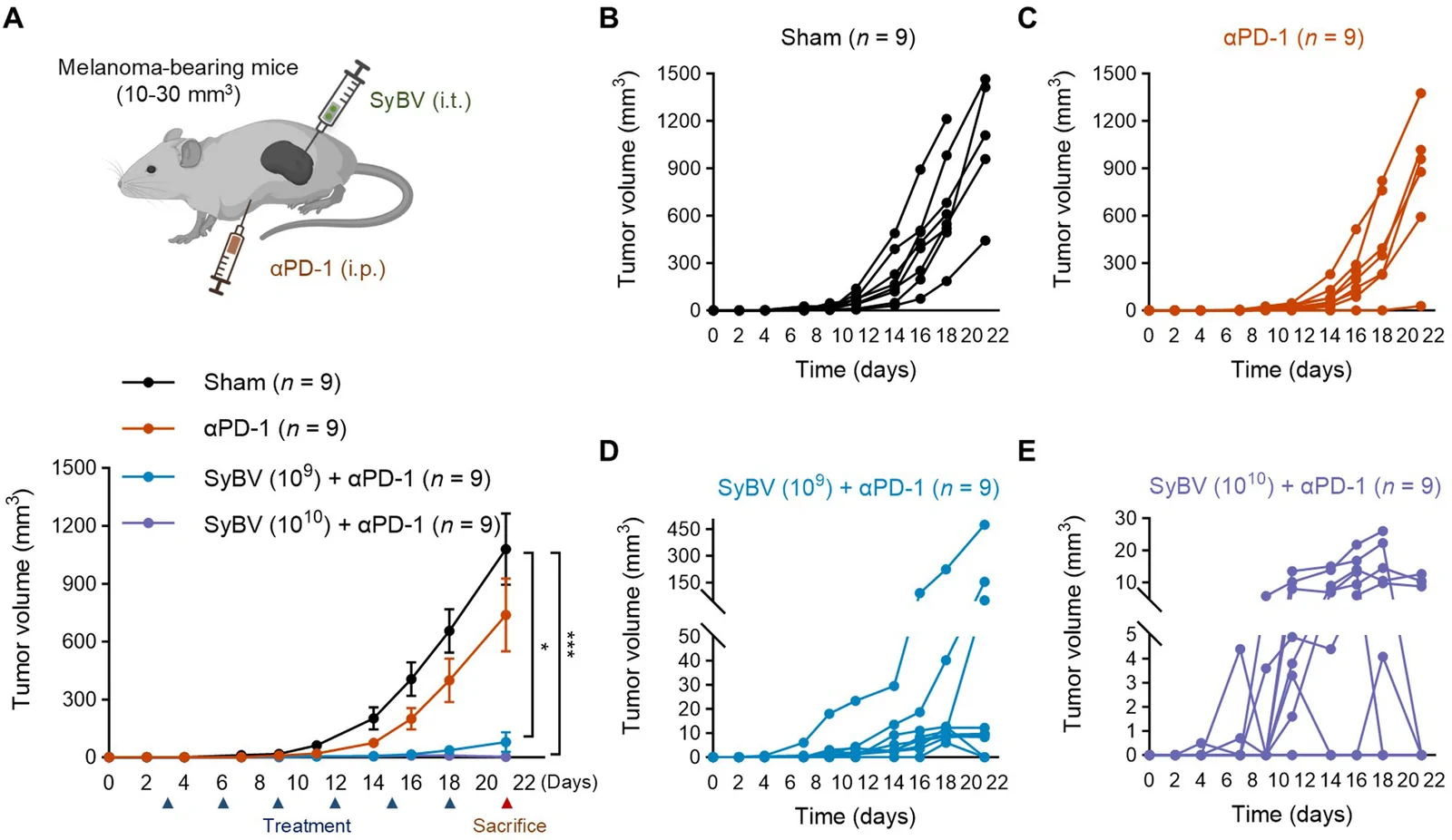
Intratumoral SyBV immunization results in complete regression of anti-PD-1-resistant melanoma. (A) Study design for therapeutic immunization with SyBV (top panel). Mice were i.t. immunized with SyBV (10^9^ or 10^10^) six times with 3-day intervals between injections following B16F10 inoculation. An anti-PD-1 antibody (100 µg) was given i.p. 1 day prior to immunization with SyBV. Tumor growth was monitored every 2-3 days (bottom panel). *n* = 9/group. Data are presented as the mean ± SEM. ^*^P < 0.05, ^***^P < 0.001 by Kruskal-Wallis analysis of variance with Dunn’s multiple comparison test; (B-E) Each individual tumor growth curve [(B) sham; (C) anti-PD-1 antibody; (D) SyBV (10^9^) plus anti-PD-1 antibody; (E) SyBV (10^10^) plus anti-PD-1 antibody] of mice shown in (A) (*n* = 9/group). Created with BioRender (2026). SyBV: Synthetic bacterial vesicle; PD-1: programmed cell death protein 1; i.t.: intratumoral; i.p.: intraperitoneal; SEM: standard error of the mean.

### Local administration of SyBV suppresses the growth of non-injected tumors

We have hypothesized that intratumoral administration of SyBV might elicit systemic effects that modulate the growth of distal, non-injected tumor tissues. To test this, mice were engrafted with B16F10 melanoma cells on both the left and right flanks, and only one tumor was treated locally with two different doses of SyBV [Figure 3A]. The growth of the injected primary tumor was shown in Figure 2. Remarkably, a significant reduction was observed in the untreated contralateral tumors at the higher dose of SyBV plus anti-PD-1 (10^10^; Figure 3A). Although distal tumors exhibited a notable inhibition (approximately 50%-60% reduction; *P* < 0.01 compared to sham), this inhibition was slightly weaker than the potent suppression at the directly treated tumor sites [Figure 2]. In contrast, systemic administration of anti-PD-1 antibodies alone had no measurable effects on either injected or contralateral tumors. We also confirmed significantly elevated serum IgG antibody levels against tumor-associated proteins in SyBV-immunized mice (approximately 3-fold increase; *P* < 0.01; Figure 3B), which may partly explain the systemic anti-tumor effects observed at distal sites. These findings highlight the potential of SyBV plus anti-PD-1 as a local immunotherapeutic agent with broader systemic efficacy. However, the precise mechanisms underlying this effect remain to be elucidated.

**Figure 3:**
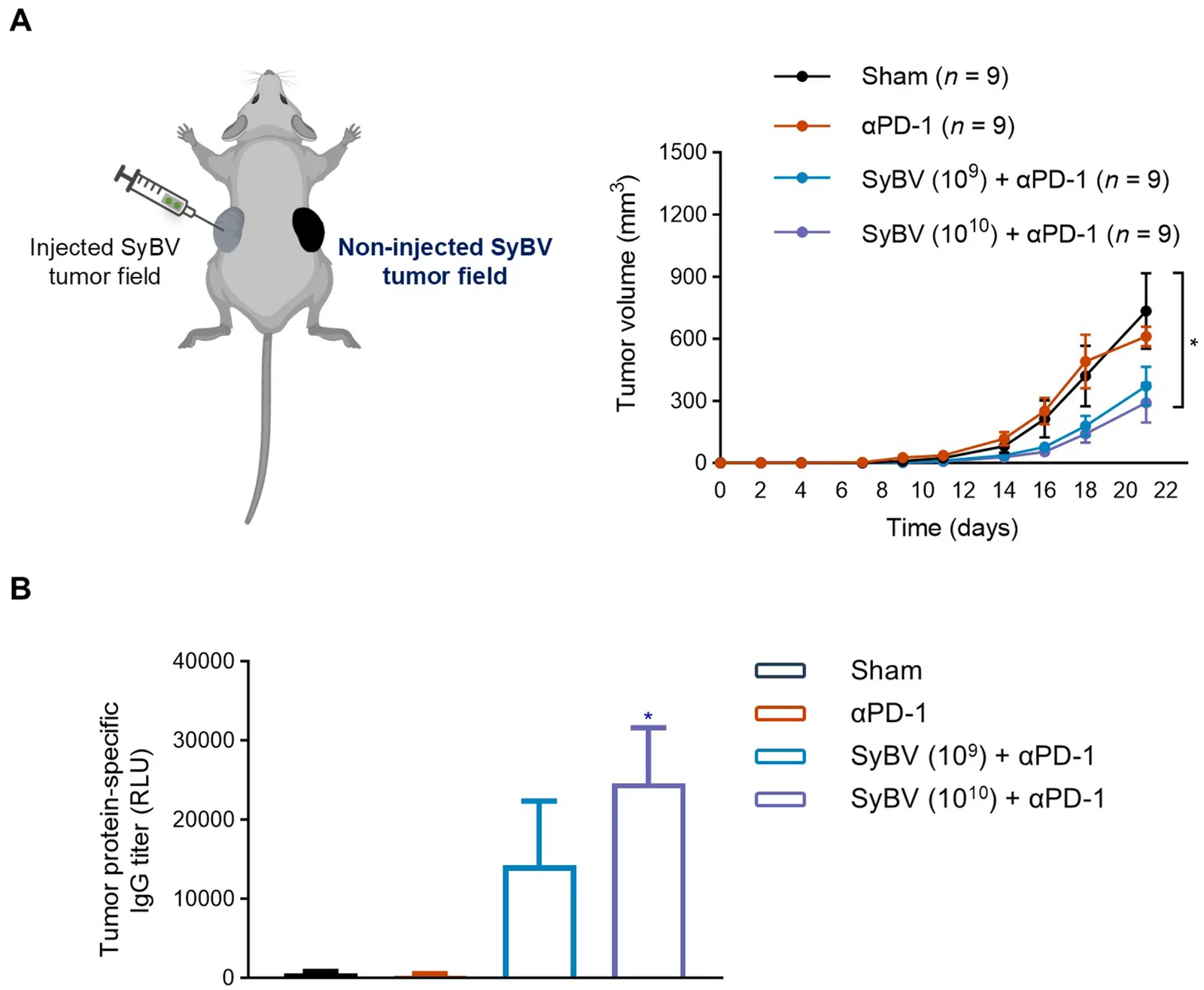
Impact of local SyBV treatment on distant melanoma growth. (A) SyBV (10^9^ or 10^10^) were intratumorally injected into the left-side melanoma six times with 3-day intervals between injections after B16F10 cells were implanted bilaterally (left panel). An anti-PD-1 antibody (100 µg) was given intraperitoneally 1 day prior to immunization with SyBV. The growth curve of non-injected tumors (right-side melanoma) in mice treated with SyBV in combination with anti-PD-1 therapy is shown (right panel). *n* = 9/group; (B) Tumor protein-specific IgG levels in blood on day 21 from mice immunized with SyBV (*n* = 5/group). Data are presented as the mean ± SEM. ^*^P < 0.05 by Kruskal-Wallis analysis of variance with Dunn’s multiple comparison test *vs*. the sham group. Created with BioRender.com (2026). SyBV: Synthetic bacterial vesicle; PD-1: programmed cell death protein 1; IgG: immunoglobulin G; SEM: standard error of the mean.

### Small early-stage tumors are more sensitive to SyBV treatment

Since early intervention significantly improves cancer prognosis^[15]^, we examined whether SyBV efficacy depends on initial tumor volume. Mice were inoculated with B16F10 melanoma and grouped based on tumor size at treatment onset: small tumors (10-30 mm^3^) and larger tumors (50-100 mm^3^). SyBV plus anti-PD-1 induced near-complete inhibition of tumor growth in mice with small, palpable tumors (*P* < 0.001 compared to sham), whereas efficacy was less in larger tumors, indicating greater sensitivity at early stages (*P* < 0.01 for small *vs*. large tumors; Figure 4). However, when treatment was initiated in mice with larger tumors, SyBV plus anti-PD-1 still achieved approximately 50% inhibition of tumor growth by day 10 of treatment (*P* < 0.05 compared to sham). These results demonstrate that the combination retains potent anti-tumor activity across tumor stages, with effective tumor reduction observed in both small and more established tumors.

**Figure 4:**
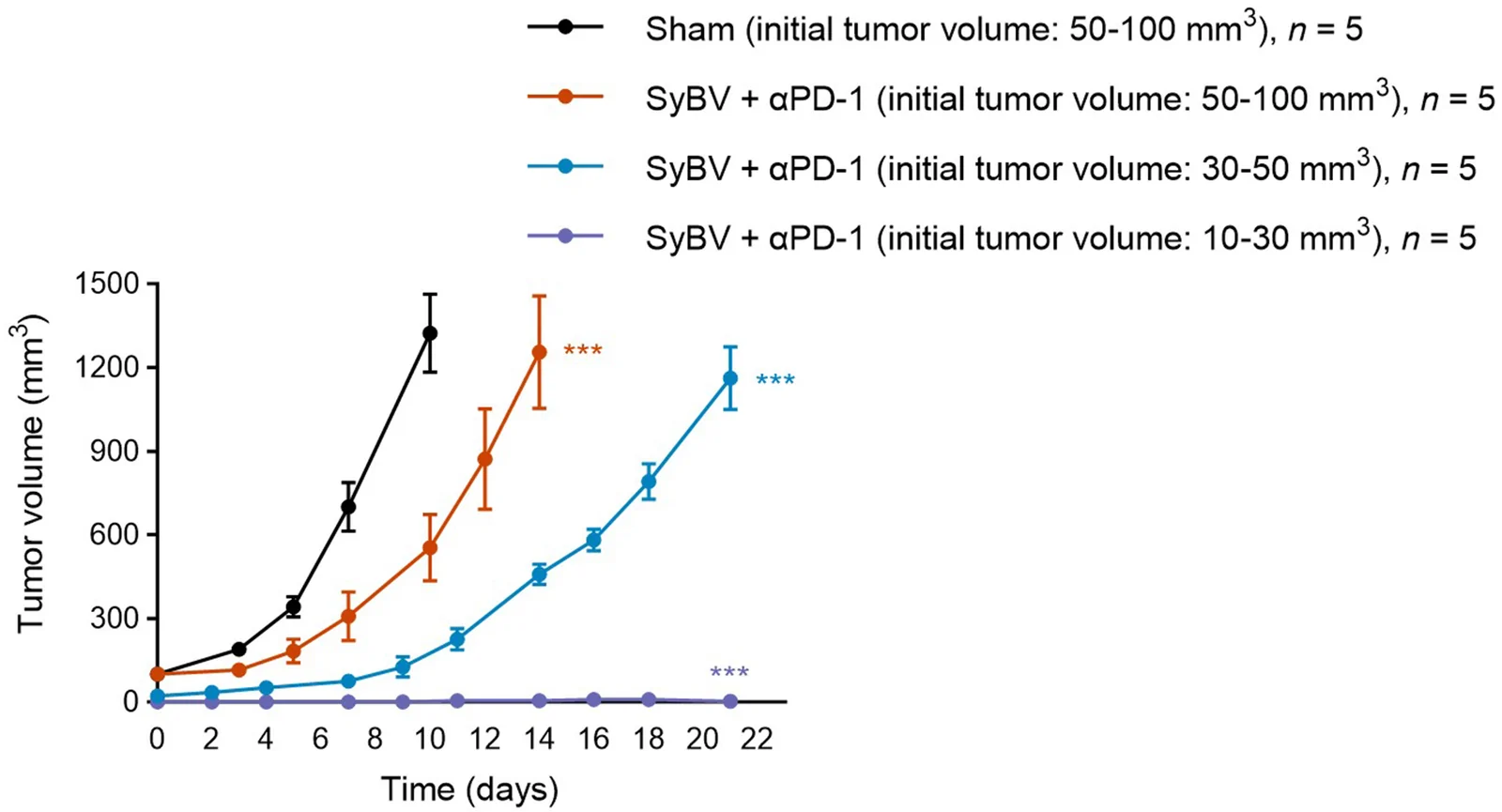
SyBV treatment is more effective in small early-stage melanoma. Tumor growth curve of mice with small (10-30 mm^3^) *vs*. larger (50-100 mm^3^) initial melanoma sizes intratumorally immunized with SyBV (10^10^). *n* = 5/group. An anti-PD-1 antibody (100 µg) was given intraperitoneally 1 day prior to immunization with SyBV. Data are presented as the mean ± SEM. ^***^P < 0.001 by two-way ANOVA with Tukey’s post-test *vs*. the sham group at the indicated time points (Day 7 shown). SyBV: Synthetic bacterial vesicle; PD-1: programmed cell death protein 1; SEM: standard error of the mean; ANOVA: analysis of variance.

### Anti-tumor activity of local SyBV administration in multiple mouse tumor models

We next evaluated whether SyBV plus anti-PD-1 exhibit broad therapeutic potential across different tumor types. To address this, we employed four syngeneic mouse tumor models, MC38 (colon), EMT6 (breast), LL/2 (Lewis lung), and RENCA (kidney), for *in vivo* experiments. Among the tested models, colon cancer showed a greater response than the other tumor types, with the treatment resulting in over 50% growth inhibition (*P* < 0.01 compared to sham; Figure 5A). Breast cancer showed a more moderate but significant reduction in growth, indicating partial sensitivity of this type of tumor (*P* < 0.05; Figure 5B). In contrast, Lewis lung and kidney tumor models showed minimal response to SyBV treatment [Figure 5C and D]. These observations highlight the selective efficacy of SyBV across certain tumors types. Furthermore, SyBV plus anti-PD1 might not be sufficient to induce robust anti-tumor effects in all tumor types tested.

**Figure 5:**
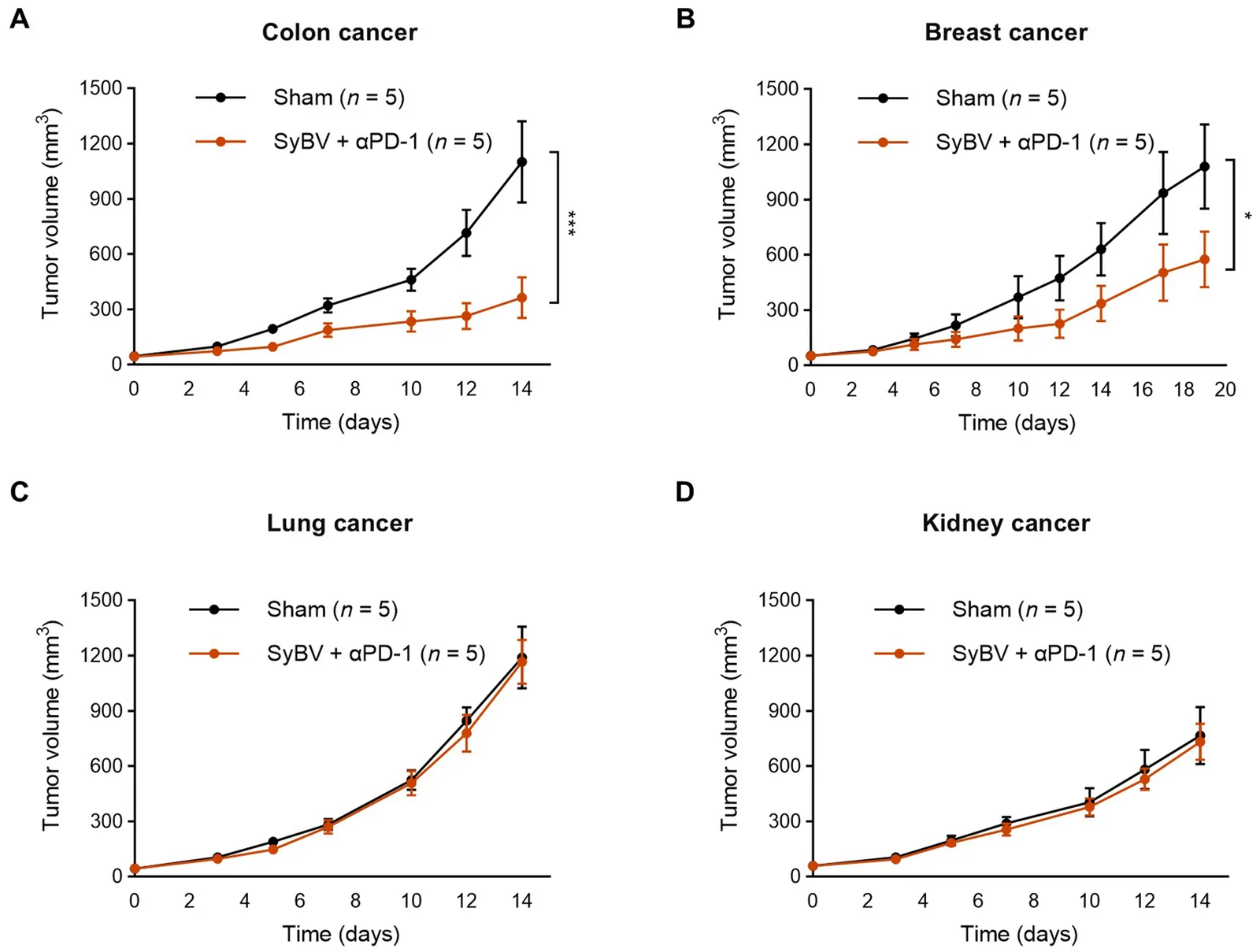
Efficacy of local SyBV administration in multiple mouse tumor models. Mice were intratumorally immunized with SyBV (10^10^) six times at 3-day intervals following inoculation with MC38 (colon cancer; A), EMT6 (breast cancer; B), LL/2 (Lewis lung cancer; C), or RENCA (kidney cancer; D) cells. An anti-PD-1 antibody (100 µg) was given intraperitoneally 1 day prior to immunization with SyBV. Tumor growth was monitored every 2-3 days. *n* = 5/group. Data are presented as mean ± SEM. ^*^P < 0.05, ^***^P < 0.001 by two-way ANOVA with Tukey’s post-test *vs*. the sham group. SyBV: Synthetic bacterial vesicle; PD-1: programmed cell death protein 1; MC38: murine colon adenocarcinoma cell line; EMT6: murine mammary carcinoma cell line; LL/2: Lewis lung carcinoma cell line; RENCA: renal adenocarcinoma cell line; SEM: standard error of the mean; ANOVA: analysis of variance.

## DISCUSSION

The present study demonstrates that local administration of SyBV effectively suppresses tumor growth across multiple preclinical cancer models, even in the absence of exogenous tumor antigens. Our results build on previous work establishing the safety and immunogenicity of SyBV^[11]^ and extend their therapeutic relevance by showing that intratumoral injection alone can elicit potent anti-tumor immunity. Importantly, we observed robust tumor regression in the poorly immunogenic B16F10 melanoma model, which is known to be resistant to immune checkpoint blockade. These findings suggest that the SyBV plus anti-PD-1 combination therapy can convert “cold” tumors into immune-responsive tumors, positioning it as a promising immunostimulatory agent that can overcome resistance to existing therapies. Importantly, the treatment was most effective in melanoma, with some efficacy also in colon- and breast cancer, but without any evident effects in lung- and kidney cancer models.

Previous studies by Caproni *et al.* and Chen *et al.* demonstrated the anti-tumor efficacy of *E. coli*-derived OMV^[14,16]^. However, the SyBV platform used in the present study is fundamentally distinct, exhibiting a cleaner vesicle composition enriched in functional membrane proteins and reduced cytosolic and toxin contaminants^[11]^. This compositional difference confers a non-toxic profile and favorable properties for clinical translation, while also enabling higher vesicle yields despite a more complex purification process. Importantly, despite these differences, SyBV retain immunostimulatory activity comparable to conventional OMV^[11]^, combining safety with practical advantages in production. Further, the SyBV are efficient in inducing crucial interleukin (IL)-12 production from dendritic cells^[11]^. While Caproni *et al.* employed the same melanoma model and intratumoral administration^[14]^, direct comparative studies between conventional OMV and SyBV are warranted to clearly define the advantages of our vesicular platform, particularly considering their demonstrated safety. Notably, although Chen *et al.* improved safety using an LPS-mutant *E. coli* strain and applied intravenous administration^[16]^, the present study demonstrates anti-tumor efficacy using intratumoral SyBV in combination with anti-PD-1 without requiring additional exogenous tumor antigens, highlighting both the importance of comparing administration routes and the practical advantages of a simplified vesicle composition.

The reduced inflammatory and toxic profiles of SyBV compared to natural OMV appear closely linked to their defined molecular composition. In particular, the absence of nucleic acid components in SyBV [Supplementary Figure 1E], together with the retention of LPS [Supplementary Figure 1F and G], suggests that the non-toxic properties of the SyBV are not solely determined by LPS content. Instead, the pro-inflammatory phenotype is likely mediated by a combination of vesicular-associated components, including both LPS and nucleic acids. Importantly, SyBV preserve Toll-like receptor (TLR)-4 signaling, consistent with surface-associated LPS, while showing diminished activation of nucleic acid-sensing TLRs, including TLR3, TLR7, TLR8, and TLR9^[11]^. This selective pattern of receptor engagement indicates that nucleic acid cargo plays a critical role in amplifying pro-inflammatory signaling in OMV. The modulation of these pathways in SyBV provides a mechanistic framework for achieving a balanced immune response with improved safety.

SyBV administration into one tumor not only inhibited local tumor growth but also slowed the progression of an untreated contralateral tumor. Such an abscopal-like effect is highly desirable in cancer immunotherapy, as it can potentially target micrometastatic lesions that are not accessible to direct treatment^[17]^. In this study, we observed increased IgG antibodies against tumor proteins in SyBV-immunized mice, indicating activation of the humoral immune response [Figure 3B]. Although circulating IgG may contribute to the observed reduction in contralateral tumor growth, IgG alone is insufficient to fully demonstrate an adaptive anti-tumor response. More targeted cellular functional assays would be needed to provide deeper mechanistic insight. Nevertheless, we previously demonstrated that SyBV induced a cellular adaptive immune response by promoting dendritic cell activation through multiple TLRs and increasing infiltration of CD4⁺ T cells, CD8⁺ T cells, and natural killer (NK) cells in both tumor tissue and lymph nodes, likely contributing to suppression of the untreated contralateral tumor^[11]^. In particular, our previous study specifically confirmed tumor-specific cytotoxic T lymphocyte responses, which play a key role in promoting tumor cell death^[11]^. Based on these established findings, it is reasonable to anticipate that similar cellular responses occur in the current study, providing mechanistic support for the measured IgG responses as part of a broader adaptive immune response.

Our data also reveal that SyBV plus anti-PD-1 treatment is most effective when initiated in smaller tumors, with early intervention leading to near-complete inhibition of tumor growth. Although previous studies have shown that smaller tumors tend to respond more readily to therapy^[18]^, our findings extend this understanding to bacterial vesicle-based cancer immunotherapy, highlighting its potential translational relevance. This size-dependent efficacy is consistent with the general observation that early-stage tumors often exhibit a less immunosuppressive microenvironment^[19]^ and are therefore more amenable to immune-based therapies. Nevertheless, even in larger, more established tumors, SyBV retained significant inhibitory activity, indicating their potential for clinical application in patients with more advanced disease. To optimize SyBV as a therapeutic strategy for established malignancies, further investigation is required to refine dosing parameters, treatment frequency, and administration intervals, and to identify rational combination regimens with other cancer therapies.

Beyond melanoma, SyBV exhibited broad anti-tumor activity in colon and breast cancer models but demonstrated minimal efficacy in lung and kidney cancer models. Investigating the mechanistic basis of these differential responses could provide valuable insights and represents an important direction for future research, although this is beyond the scope of the present study. Nevertheless, this variability may reflect intrinsic differences in tumor heterogeneity, immune cell infiltration, and the immunosuppressive characteristics of the tumor microenvironment across cancer types. In particular, neoantigens derived from mutated tumor cells are likely critical for eliciting robust anti-tumor responses^[20]^. Consequently, the lack of significant neoantigen quantities in any tumor, or their insufficient presentation by dendritic cells, may contribute to resistance to cancer immunotherapy. Because tumor cells can release extracellular vesicles carrying tumor-specific antigens^[21,22]^, combining SyBV with neoantigen-enriched vesicles may represent a promising strategy to overcome its current limitations and expand its efficacy across a wider spectrum of tumor types. Moreover, it is important to note that the subcutaneous tumor models used in this study do not fully replicate the native tumor microenvironment, including tissue-specific stromal, immune, and vascular interactions that can critically influence therapeutic responses. This limitation may contribute to the differential efficacy observed across tumor types and could also lead to exaggerated immunotherapy effects, such as abscopal responses, that are not typically seen in clinical settings. Therefore, while our findings provide valuable insights into the anti-tumor potential of SyBV, caution is warranted when extrapolating these results to more physiologically relevant tumor models or clinical scenarios.

In summary, our study establishes SyBV as a potent, safe, and versatile platform for local cancer immunotherapy. By leveraging endogenous tumor antigens, SyBV can induce robust local and systemic anti-tumor immunity, sensitize resistant tumors to checkpoint blockade, and significantly inhibit tumor growth across multiple cancer types. Despite the promising anti-tumor efficacy of SyBV demonstrated in our study, several limitations should be acknowledged. As detailed above, first, the observed efficacy varied across tumor types, suggesting that intrinsic differences in tumor biology and immune responsiveness may influence outcomes. Second, the reliance on intratumoral administration does not address how SyBV might perform with systemic delivery, which could be an interesting alternative for treating metastatic or inaccessible tumors. Third, the preclinical models employed, including subcutaneous xenografts, do not fully replicate the complexity of human tumors, including tissue-specific microenvironments, vascular networks, and immune landscapes, which may affect both efficacy and safety in clinical settings. Fourth, the timing of intervention emerged as an important factor, with earlier treatment correlating with better outcomes, highlighting the need for optimized dosing schedules in future translational studies. Finally, while SyBV offers practical advantages over conventional vesicles, additional comparative studies and long-term evaluations are necessary to fully define their therapeutic window, potential off-target effects, and combination strategies with existing immunotherapies. Addressing these points in future studies will be critical to expanding the clinical applicability of SyBV-based immunotherapy and to guiding rational strategies to overcome resistance across diverse tumor types.

## DECLARATIONS

### Availability of data and materials

The data and materials supporting the findings of this study are available upon reasonable request from the corresponding authors.

### Copyright

© The Author(s) 2026.
