# Supplementary material for: Bacterial membrane vesicles as effective immunotherapeutic agents in melanoma, colon, and breast cancer
Source: Extracell Vesicles Circ Nucl Acids. 2026 Jul 6;7(3):1043–55. doi: 10.20517/evcna.2026.07 (PMC13678171; doi:10.20517/evcna.2026.07)
Supplement: Supplementary file 1 [file evcna-7-3-1043-SupplementaryMaterials.pdf]

## Supplementary Materials

**Bacterial membrane vesicles as effective immunotherapeutic agents in melanoma, colon, and breast cancer**

**Jan Lötvall[1], Negar Ordouzadeh[1,2], Kyong-Su Park[1]**

[1]Krefting Research Centre, Institute of Medicine, Sahlgrenska Academy, University of Gothenburg, Gothenburg 40530, Sweden.
[2]Exocure Sweden AB, Gothenburg 41126, Sweden.

**Correspondence to:** Dr. Kyong-Su Park, Krefting Research Centre, Institute of Medicine, Sahlgrenska Academy, University of Gothenburg, Gothenburg 40530, Sweden. E-mail: kyong-su.park@gu.se

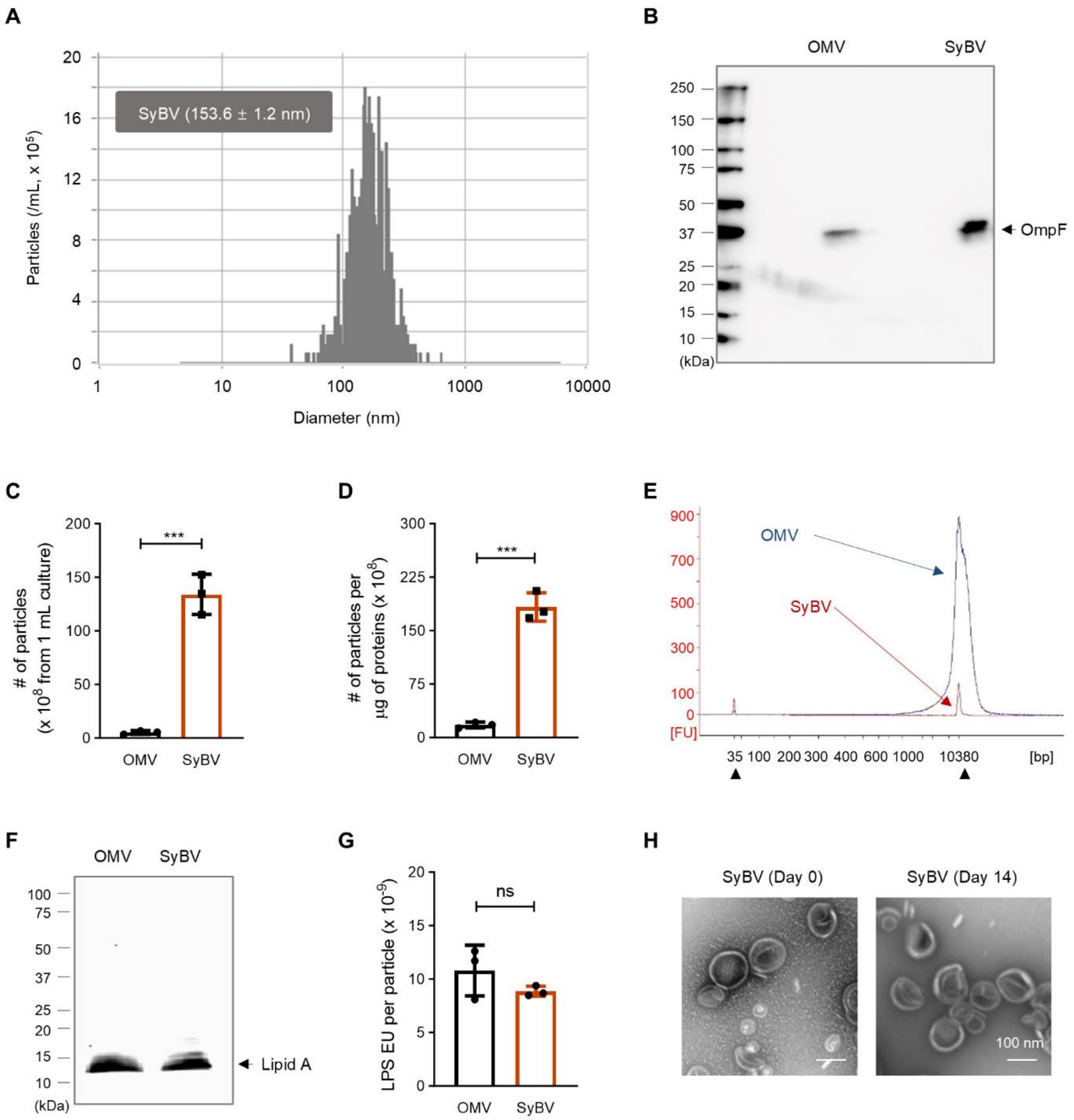


**Supplementary Figure 1.** Characterization of *E. coli*-derived SyBV. (A) Size distribution of SyBV measured by nanoparticle tracking analysisp; (B) Western blot analysis of OMV ($10^9$) and SyBV ($10^9$) with anti-OmpF antibody; (C and D) The number of particles derived from 1 mL bacterial culture (C) and the number of particles per one microgram of vesicular proteins (D) from OMV and SyBV. $n = 3$ / group. Data are presented as the mean ± SEM. $^{***}P < 0.001$ by unpaired two-tailed Student's *t*-test; (E) Representative electropherograms of DNA molecules isolated from SyBV in comparison to those from OMV. The filled triangles indicate internal markers; (F) Western blot analysis of OMV ($10^9$) and SyBV ($10^9$) with anti-lipid A antibody; (G) The LPS quantity as determined by the Limulus Amebocyte Lysate (LAL) assay. EU, enzyme unit. $n = 3$ / group. Data are presented as the mean ± SEM. ns, not significant by unpaired two-tailed Student's *t*-test; (H) TEM images comparing fresh SyBV (Day 0) with frozen SyBV after 14 days of storage. Scale bars, 100 nm.

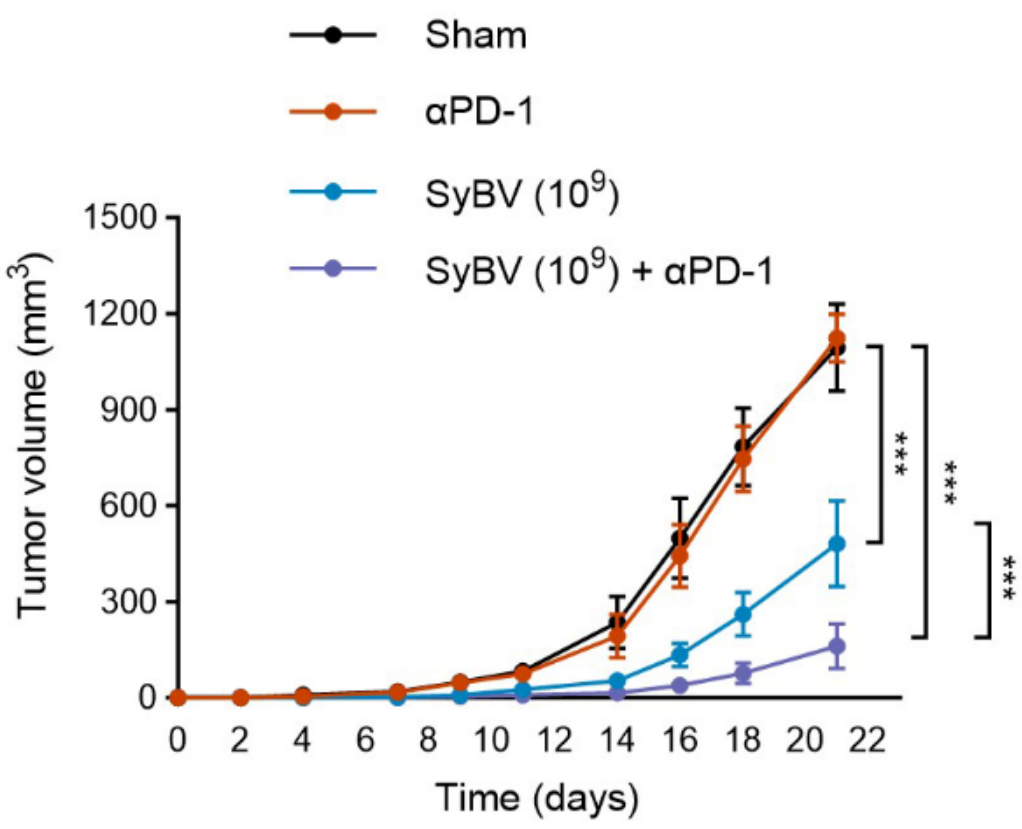


**Supplementary Figure 2.** SyBV treatment synergizes with anti-PD-1 therapy to inhibit melanoma growth in mice. Mice were intratumorally immunized with SyBV ($10^9$) six times at 3-day intervals following B16F10 inoculation. Anti-PD-1 antibody (100 µg) was intraperitoneally given one day prior to immunization with SyBV. The tumor growth was monitored every 2 or 3 days. $n = 5$ / group. Data are presented as the mean ± SEM. $^{***}P < 0.001$ by two-way ANOVA with Tukey's post test.

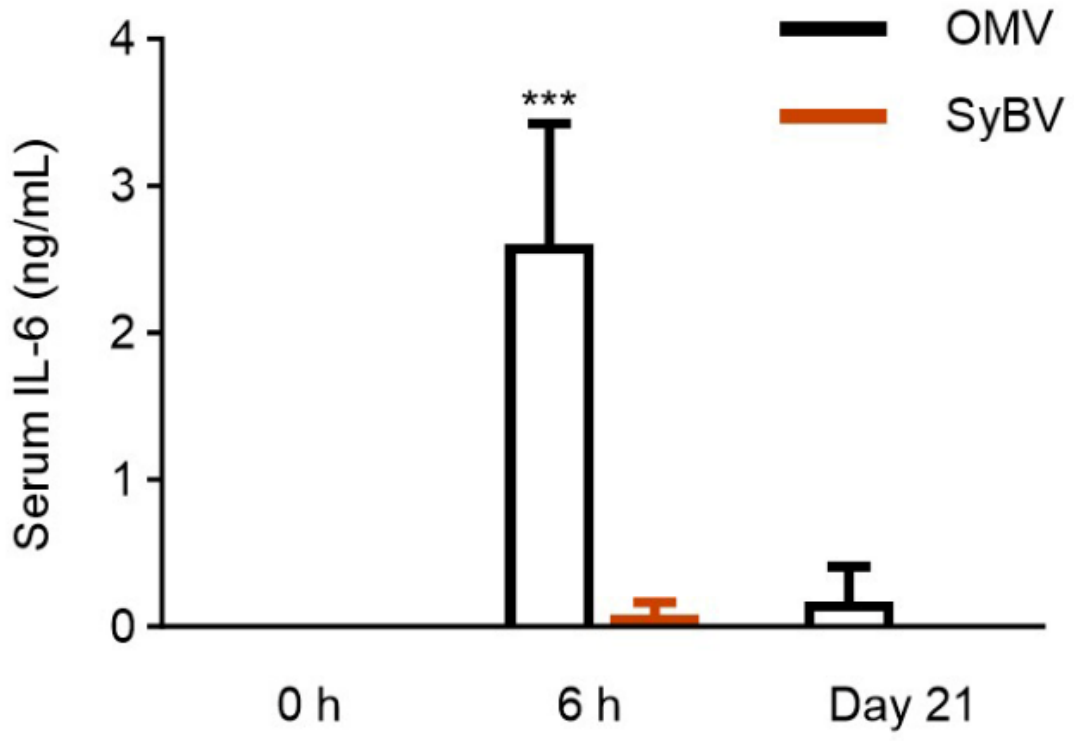


**Supplementary Figure 3.** SyBV do not induce a systemic toxicity during immunization. Kinetics of serum interleukin (IL)-6 concentrations in mice administered with OMV ($10^{10}$) or SyBV ($10^{10}$). *n* = 4 / group. Data are presented as the mean ± SEM. $^{***}P < 0.001$ by two-way ANOVA with Tukey's post test versus 0 h group.

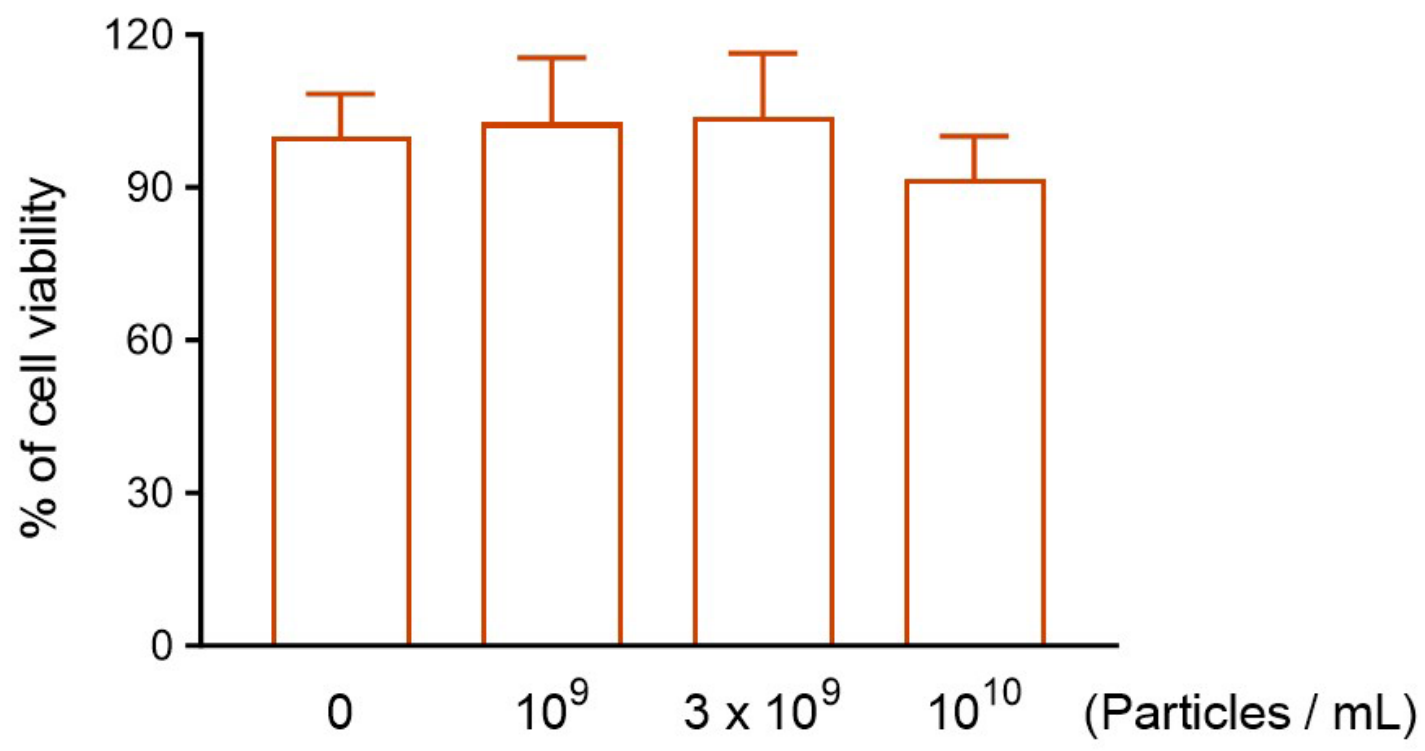


**Supplementary Figure 4.** SyBV do not directly affect the growth of tumor cells. The B16F10 cells were treated with various concentrations of SyBV for 24 h and cell viability was assessed by MTT assay. Results are expressed in percentage of control. $n = 3$ / group. Data are presented as the mean ± SEM.
